# Beyond recurrence detection: advancing TPUS applications in hemorrhoidal disease through context-specific protocols and AI integration

**DOI:** 10.1007/s00384-025-04988-6

**Published:** 2025-08-27

**Authors:** Hengqing Gao, Lingyu Liu, Yuting Xi

**Affiliations:** 1 Department of Anorectal Diseases II, Zigong Hospital of Traditional Chinese Medicine, No. 336, Huixing Road, Ziliujing District, Zigong, 643010 Sichuan China; 2 Chengdu University of Traditional Chinese Medicine, Chengdu, 640000 Sichuan China

Dear Editor:

We read with considerable interest the scholarly exchange between Gravante et al. and Schiano di Visconte concerning the clinical utility of transperineal ultrasound (TPUS) in the follow-up management of hemorrhoidal disease (HD), along with Gravante et al.’s comprehensive rebuttal to the methodological critique [1–3]. This academic discourse not only highlights the indispensable role of rigorous scientific debate in advancing innovative diagnostic modalities but also prompts critical examination of strategies for translating preliminary research evidence into clinically robust practice protocols. In the present commentary, we seek to elucidate two underinvestigated aspects: (1) the differential utility of TPUS across hierarchical healthcare settings and (2) the systematic incorporation of patient-reported outcome measures to optimize clinical relevance.

Gravante et al. underscore the accessibility of TPUS, highlighting its reliance on standard ultrasonographic equipment that is ubiquitously available even in non-specialized clinical settings [1]. While this represents a significant logistical advantage, the diagnostic utility of TPUS may exhibit substantial heterogeneity across different tiers of healthcare delivery. In primary care or resource-limited rural clinics—where endoscopic capabilities are often constrained—TPUS could function as an efficient triage modality, facilitating the identification of patients warranting specialist referral. Conversely, in tertiary care centers, TPUS may assume a complementary role to endoanal ultrasound (EAUS), providing a non-compressive physiological baseline prior to invasive diagnostic evaluations.

Notably, Gravante et al. deliberately abstained from direct comparisons between TPUS and EAUS to mitigate potential confounding from vascular compression artifacts [1]. However, future investigations might evaluate a staged diagnostic algorithm: initial TPUS screening to exclude recurrence (evidenced by scattered vascular patterns), followed by selective EAUS application only in cases exhibiting focal vascular patterns to delineate anatomical severity. Such a stratified approach could enhance resource allocation efficiency in specialized clinical environments while maintaining diagnostic accuracy.

Future multicenter investigations should implement care-setting stratification (primary vs. tertiary) to establish context-specific TPUS protocols, including primary care referral thresholds or tertiary center EAUS integration. This aligns with broader demands for TPUS application research across healthcare scenarios, as evidenced by anal pathology studies [4, 5].

While existing work emphasizes TPUS-endoscopic recurrence correlation, its symptom association remains underexplored. Gravante et al. reported nonsignificant vascular pattern-symptom relationships [1], whereas Schiano di Visconte emphasized this diagnostic discrepancy’s clinical implications [2]. This raises a pivotal question: Do vascular patterns without endoscopic recurrence predict symptom progression at 6 to 12 months? Affirmative findings could redefine TPUS from recurrence detection to symptomatic deterioration prediction.

Prospective studies should incorporate longitudinal Patient-Reported Outcome Measures (PROMs) with serial TPUS evaluations, correlating vascular evolution with symptom trajectories. This would determine whether TPUS findings possess prognostic value independent of endoscopic confirmation.

To advance beyond accuracy debates, we advocate AI-endoscopy integration. Convolutional neural networks could standardize Doppler parameter quantification (flow velocity/vessel density), reducing operator variability [6]. TPUS-endoscopy fusion may enable targeted biopsy of sonographically suspicious zones, replacing random sampling with hypothesis-driven validation. This “US-AI-Endoscopy Triad” would facilitate multidimensional assessment, combining TPUS’s hemodynamic data with structural findings.

The Gravante-Schiano discourse establishes TPUS’s foundational role in HD management. Refining context-specific applications, PROM integration, and imaging standardization will transition TPUS from validation to optimization—transforming it from investigational tool to essential proctologic practice.

## Data Availability

No datasets were generated or analysed during the current study.
